# Risk factor analysis for bone marrow histiocytic hyperplasia with hemophagocytosis: an autopsy study

**DOI:** 10.1007/s00428-014-1592-8

**Published:** 2014-05-23

**Authors:** Kunihiro Inai, Sakon Noriki, Hiromichi Iwasaki, Hironobu Naiki

**Affiliations:** 1 Division of Molecular Pathology, Department of Pathological Sciences, University of Fukui, Eiheiji Fukui, 910-1193 Japan; 2 Division of Tumor Pathology, Department of Pathological Sciences, University of Fukui, Eiheiji Fukui, 910-1193 Japan; 3 Division of Hematology and Oncology, Department of General Medicine, University of Fukui, Eiheiji Fukui, 910-1193 Japan

**Keywords:** Bone marrow, Hematological diseases, Histiocytic hyperplasia with hemophagocytosis, Inflammation, Inflammatory cytokine, Sepsis

## Abstract

The excessive release of inflammatory cytokines occasionally induces life-threatening hemophagocytosis referred to as hemophagocytic syndrome (HPS). A similar condition, histiocytic hyperplasia with hemophagocytosis (HHH), is often seen in bone marrow collected during autopsy. Unlike HPS, the pathogenesis of HHH remains unclear. Therefore, we performed a clinicopathological analysis of HHH from 70 autopsy cases at the University of Fukui Hospital. HHH was detected in 29 of 70 autopsies (41.4 %) and was significantly complicated with hematological diseases (*p* < 0.05) and sepsis (*p* < 0.05). The percentage of macrophages in bone marrow (BM) nucleated cells was significantly increased in HHH (*p* < 0.001). Data from medical records indicated no significant changes, except for the minimum values of white blood cell counts (*p* < 0.05) and platelet counts (*p* < 0.05) in HHH patients as compared with non-HHH patients. Concentrations of inflammatory mediators including IL-1β, IL-6, and IL-8 were significantly increased in HHH patients. Multivariate risk factor analysis identified hematological diseases (odds ratio (OR), 11.71), ≥15 % BM macrophages (OR, 9.42), sepsis (OR, 7.77), and high serum IL-6 levels (OR, 1.00) as independent risk factors for HHH. HHH with hypocellular BM, the most aggressive form of HHH, was recognized in 8 of 29 HHH patients and was associated with ≥25 % BM macrophages (*p* < 0.001), leukocytopenia (*p* < 0.05), and high IL-8 levels (*p* < 0.05). None of the HHH patients fulfilled the diagnostic criteria of HPS. These findings suggest that HHH is a different entity from HPS and that it preferentially develops under conditions of excessive inflammation and its associated risks, such as hematological diseases and sepsis.

**Electronic supplementary material:**

The online version of this article (doi:10.1007/s00428-014-1592-8) contains supplementary material, which is available to authorized users.

## Introduction

The systemic release of excessive amounts of inflammatory cytokines results in severe clinical disorders such as acute respiratory distress syndrome, disseminated intravascular coagulation (DIC), septic shock, and multi-organ failure [1]. Simultaneously, the mononuclear–macrophage system is often dramatically activated [2], so that some patients develop a rare life-threatening syndrome called hemophagocytic syndrome (HPS) or hemophagocytic lymphohistiocytosis [3, 4]. The hallmark of HPS is sustained inflammatory cytokinemia due to genetic inheritance, infections, malignant lymphoma, and collagen diseases [3, 5, 6]. Patients with HPS develop persistent high fever, pancytopenia, hepatosplenomegaly, lymphadenopathy, and elevated levels of liver transaminases, bilirubin, lactate dehydrogenase, ferritin, and triglycerides [4, 7]. Histopathological examination reveals the presence of activated macrophages in hematopoietic organs such as bone marrow, liver, spleen, and lymph nodes, where they often engulf erythrocytes and hematopoietic nucleated cells [4].

Bone marrow samples from deceased patients often reveal similar findings of hemophagocytosis [2, 8]. This phenomenon, known as histiocytic hyperplasia with hemophagocytosis (HHH), can be observed in patients with sepsis, viral and non-viral infections, and advanced malignancies [8, 9]. Based on these observations, we hypothesized that the pathogenesis of HHH may be initiated by a series of cytokine storms including the release of inflammatory interferon (IFN)-γ, tumor necrosis factor (TNF)-α, interleukin (IL)-1β, IL-6, IL-10, and IL-18 cytokines, and IL-8 chemokine, as occurs in HPS [5, 6, 10]. However, it remains controversial whether the development of HHH is caused by malignant diseases [2], and the pathogenesis of HHH remains obscure. In the present study, we investigated the pathogenesis of HHH by performing a postmortem analysis of 70 patients with HHH. The primary outcome was to identify risk factors for the development of HHH, and the secondary endpoint was to analyze the inflammatory cytokine profiles that characterize HHH by using serum collected at autopsy. We found that HHH preferentially developed under conditions of excessive inflammation, including hematological diseases such as leukemia, lymphoma, and aplastic anemia and sepsis, and that its onset and severity were associated with pro-inflammatory IL-6 and IL-8, respectively.

## Materials and methods

### Patients and criteria for HHH

During the 3-year study period, we performed 87 autopsies. Seven of these cases were excluded due to the inability to collect serum because of coagulation, and ten cases were excluded because only a partial dissection was performed. The remaining 70 patients (39 men and 31 women) ranged in age from 0 to 94 years (median age, 68 years) at the time of death. Autopsies were performed at the University of Fukui Hospital and were retrospectively examined. Written informed consent was obtained from the families of each deceased patient prior to enrollment. All research protocols were approved by the ethics review board at the University of Fukui Hospital and conformed to the provisions of the Declaration of Helsinki.

The activity of hemophagocytosis was categorized though microscopic examination of bone marrow as follows: (a) mild HHH, several high-power fields must be searched to find hemophagocytosis; (b) moderate HHH, 1–3 hemophagocytic cells per high-power field; (c) severe HHH, more than 3 hemophagocytic cells per high-power field; and (d) hypoplastic HHH (hypo-HHH), severe HHH with hypocellular bone marrow. We assumed that cases showing moderate HHH to hypo-HHH represented HHH in accordance with previously described criteria [2, 8].

### Lists of inspection data

Data including age, sex, diagnosis on admission, complications, DIC, infectious disease, sepsis, duration until autopsy, and cause of death were obtained from clinical and autopsy records (Electronic supplementary material (ESM) Table 1). Mean duration until autopsy was 2.9 ± 2.6 h (1–15.5 h; median duration, 2 h) after the patient’s death. This time was similar in cases with HHH (3.4 ± 3.3 h) and with non-HHH (2.7 ± 2.0 h). Sepsis was verified by the repeated detection of microbes from ante-mortem blood cultures from patients who met the criteria of systemic inflammatory response syndrome [11, 12] or by the detection of scattered micro-abscesses with or without bacterial, fungal, or viral inclusion bodies in histopathological studies of different tissues. DIC was diagnosed based on the criteria of the Japanese Ministry of Health, Labor and Welfare [13] or from the existence of fibrin thrombi in tissue sections collected during autopsy. Shock was pathologically confirmed by detecting shock-related morphological changes including diffuse alveolar damage (shock lung), scattered focal or sub-massive necrosis around the central hepatic vein (shock liver), and/or acute tubular necrosis (shock kidney) [1]. Hepatomegaly was defined as a liver weight greater than 1,300 g (normal range in Japanese people, 900–1,300 g), and splenomegaly was defined as a spleen weight of 130 g or greater.

**Table 1:** Patient characteristics with or without HHH

Category	Subcategory	Total	HHH	No HHH	P*
Number of patients		70	29	41	
Age	Average	66.5	65.4	66.9	
	Range	0-94	52-91	0-94	
	Median	68	65	71	
Sex	Male	39	16	23	
	Female	31	13	18	
Hematological diseases		19	14	5	<0.05
Malignancy	All	49	22	27	
	Hematological	18	13	5	<0.05
	Solid	31	9	22	
Infection	All	42	22	20	
	Sepsis	18	13	5	<0.05
	Pneumonia	16	12	14	
DIC		32	18	14	
Shock		17	9	8	

*HHH* histiocytic hyperplasia with hemophagocytosis, *DIC* disseminated intravascular coagulation

*Adjusted using the Holm method for multiple testing

The maximum and minimum white blood cell (WBC) counts, minimum hemoglobin (Hb) level and platelet count (Plt), and peak serum values of aspartate aminotransferase (AST), triglyceride, ferritin, and soluble IL-2 receptor (sIL-2R) from within 100 days prior to death were collected from medical records.

### Morphological analysis and immunohistochemical staining

Thin sections of paraffin-embedded bone marrow were stained with hematoxylin and eosin. Activated macrophages were evaluated by immunohistochemical staining with primary anti-CD68 antibody (DAKO, Kyoto, Japan) and antibody against galactin-3 (Nichirei, Tokyo, Japan), a carbohydrate-binding lectin secreted by activated macrophages [14]. A rabbit polyclonal anti-CD3 antibody (DAKO) was used to determine the number of T lymphocytes. The Berlin blue stain was performed to detect intra- and extracellular iron deposition [8]. The sections were evaluated by two different pathologists who specialize in the pathophysiological analysis of infection and inflammatory diseases [15, 16].

### Cytokine analysis

Blood samples were collected from the right atrium by insertion of an 18-G needle attached to a 20-ml syringe after resection of the ribs and cardiac sac at autopsy. After clotting, the blood was spun at 1,000 × *g* for 10 min at room temperature. The separated serum was transferred to 1.5-ml tubes and stored at −80 °C until analysis. The levels of inflammatory TNF-α, IL-1β, IL-6, IL-10, and IL-12 cytokines, and IL-8 chemokine were determined by flow cytometry (FACs Calibur; Becton Dickinson, Franklin Lakes, NJ) performed with a Human Inflammation Kit (BD Biosciences, San Diego, CA) according to the manufacturer’s protocol. IFN-γ was measured by using a BD cytometric bead array (Becton Dickinson) as described before [17]. If the measured levels exceeded the upper limits of the equipment, serum was diluted with saline and then re-evaluated by the same method.

### Statistical analysis

Statistical analysis was performed by using the *χ* ^2^ test and Mann–Whitney *U* test. Pearson and Spearman correlation coefficients were calculated as appropriate. Significant associations with HHH were further analyzed by using logistic regression analysis. *P* values were adjusted by using the Holm method for multiple testing [18], and the results before and after the adjustment are shown in ESM Tables 2 and 3. Values of *p* < 0.05 were accepted as statistically significant. All statistical analyses were performed with Ekuseru-Toukei 2010 software (Social Survey Research Information, Tokyo, Japan).

## Results

### Patient characteristics with HHH

**Fig. 1:**
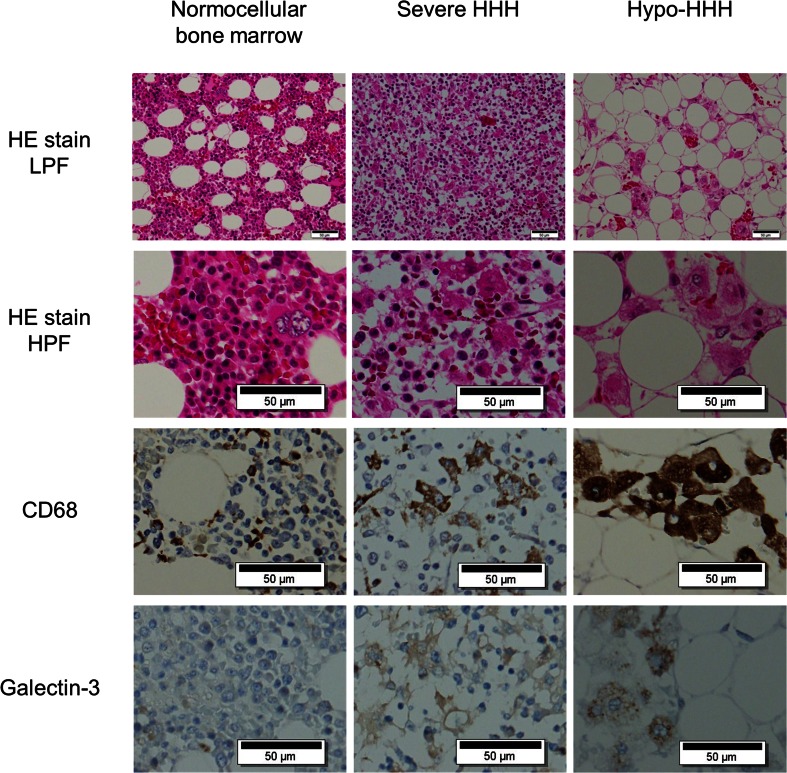
Morphological features of bone marrow at autopsy in patients with HHH. (*Top line*) Low-power magnification of bone marrow stained by hematoxylin and eosin. (*2nd line*) High-power magnification of HE-stained bone marrow. Activated macrophages engulfing erythrocytes and nucleated cells were scattered in severe HHH (*center column*) and hypo-HHH (*right column*) sections. (*3rd line*) Immunohistochemical staining with anti-CD68 antibody. Although CD68-positive macrophages, including brown deposits in the cytoplasm, were present in all bone marrow tissues, large amoebic-shaped cells were only noted in severe HHH and hypo-HHH bone marrow. (*Bottom line*) Immunohistochemical staining of galectin-3 secreted by active macrophages. Galectin-3 was expressed in bone marrow from severe HHH and hypo-HHH patients. *HHH*, histiocytic hyperplasia of hemophagocytosis; *HE*, hematoxylin and eosin; *LPF*, low-power field; *HPF*, high-power field

**Fig. 2:**
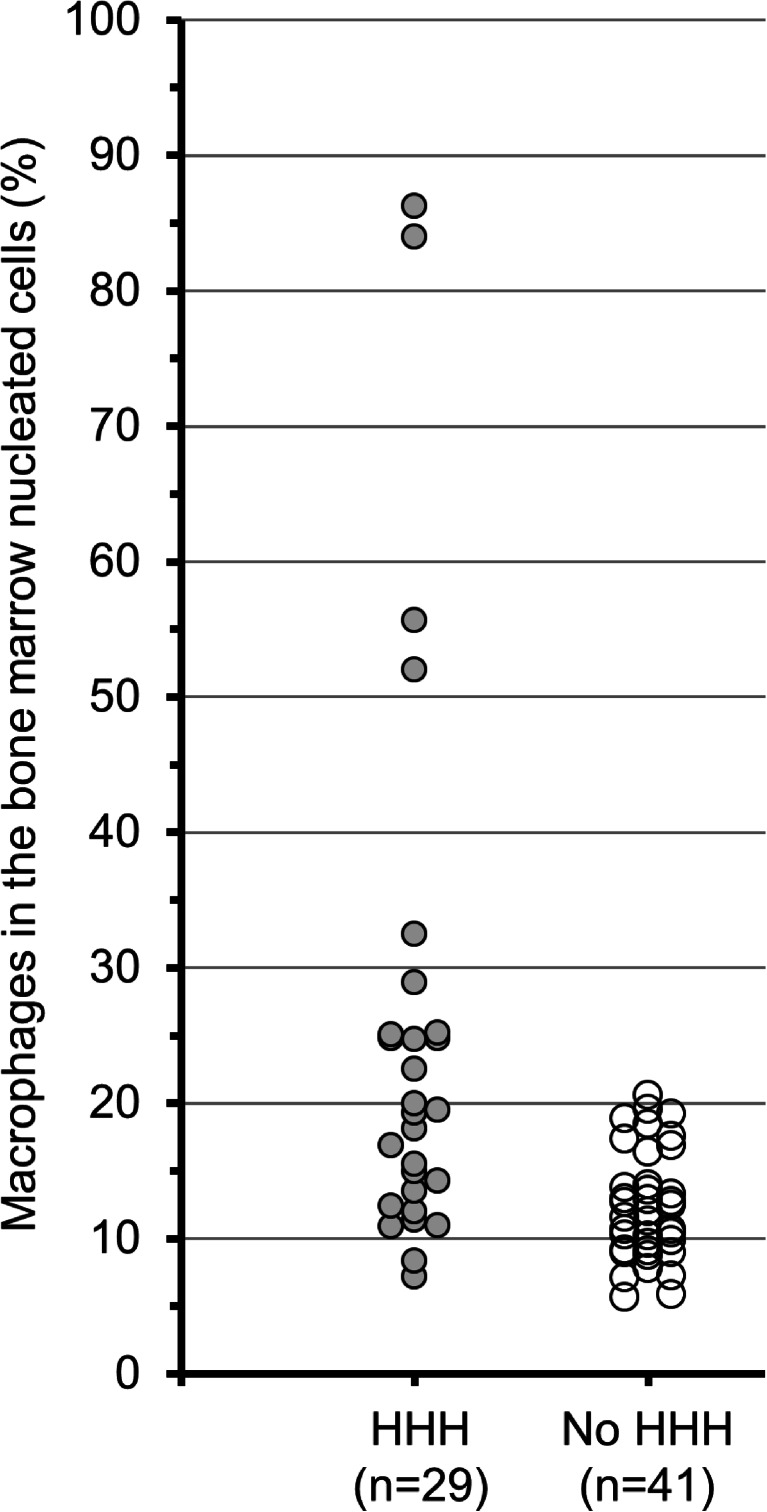
Percentage of macrophages in the bone marrow nucleated cells. The macrophages in the bone marrow were evaluated by CD68 immunohistochemical stain. Bone marrow nucleated cells were counted as Giemsa-staining positive cells. *HHH*, histiocytic hyperplasia of hemophagocytosis

Twenty-nine of 70 patients (41.4 %) were categorized as having HHH. The HHH group consisted of high-grade cases demonstrating moderate HHH (*n* = 10), severe HHH (*n* = 11), or hypo-HHH (*n* = 8). The remaining 41 patients categorized as having no HHH consisted of 14 patients with mild HHH and 27 normal cohorts. Bone marrow cellularity of the 70 patients was characterized as hypercellular (*n* = 26), slightly hypercellular (*n* = 3), normocellular (*n* = 28), slightly hypocellular (*n* = 1), and hypocellular (*n* = 12). Representative morphological features are shown in Fig. 1. CD68-expressing cells were scattered throughout all bone marrow sections. In particular, severe HHH and hypo-HHH cases contained large amebic histiocytes expressing both CD68 and galectin-3 proteins; these cells were considered to represent activated macrophages. The cytoplasm of many activated cells contained engulfed erythrocytes, nucleated cells, and/or vacuoles. The percentage of CD68-positive cells in the bone marrow nucleated cell number was significantly increased in patients with HHH than in patients without HHH (25.2 % ± 20.0 % vs. 12.1 % ± 4.0 %, *p* < 0.001) (Fig. 2). Clinical characteristics of patients with or without HHH are shown in Table 1. Hematological diseases (*p* < 0.05), hematological malignancies (*p* < 0.05), and sepsis (*p* < 0.05) were significantly more common in patients with HHH than in patients without HHH. Malignancies, pneumonia, and shock were not associated with HHH.

### Sepsis as a critical cause of death in HHH

To clarify the characteristic causes of death in HHH, the causes of death were categorized (Table 2). The following causes of death were identified in the 29 HHH patients: acute respiratory distress syndrome (ARDS)/sepsis/septic shock (*n* = 11), cancer (*n* = 6), organ failure such as congestive heart failure or liver failure (*n* = 6), pneumonia (*n* = 3), and shock other than septic shock (*n* = 3). Organ failure was the predominant cause of death in non-HHH patients, followed by shock and cancer. Death caused by sepsis/septic shock or ARDS occurred in only two non-HHH cases. The percentage was significantly lower than that of HHH cases (2/41 vs. 11/29, *p* < 0.05).

**Table 2:** Cause of the patient’s death with or without HHH

	HHH	No HHH
ARDS/sepsis/septic shock	11	2
Pneumonia/other infection	3	4
Other shock/bleeding	3	11
Cancer death/cachexia/emaciation	6	10
Organ failure/heart, respiratory, liver, or renal	6	13

*HHH* histiocytic hyperplasia with hemophagocytosis, *ARDS* acute respiratory distress syndrome

The pathogens isolated by microscopic inspection from septic HHH patients were fungi (*n* = 6; *Candida* in three patients, *Aspergillus* in two patients, and *Fusarium* in one patient), cocci (*n* = 6), bacilli (*n* = 3), and cytomegaloviruses (CMV) (*n* = 3). Three different pathogens (fungus, CMV, and cocci) were isolated from two patients, and a female patient with microscopic polyangiitis had both *Candida* and *Aspergillus* infections. In contrast to HHH patients, three patients with cocci and one patient with bacilli were found among the 41 non-HHH patients.

### High serum inflammatory cytokine levels in HHH

**Fig. 3:**
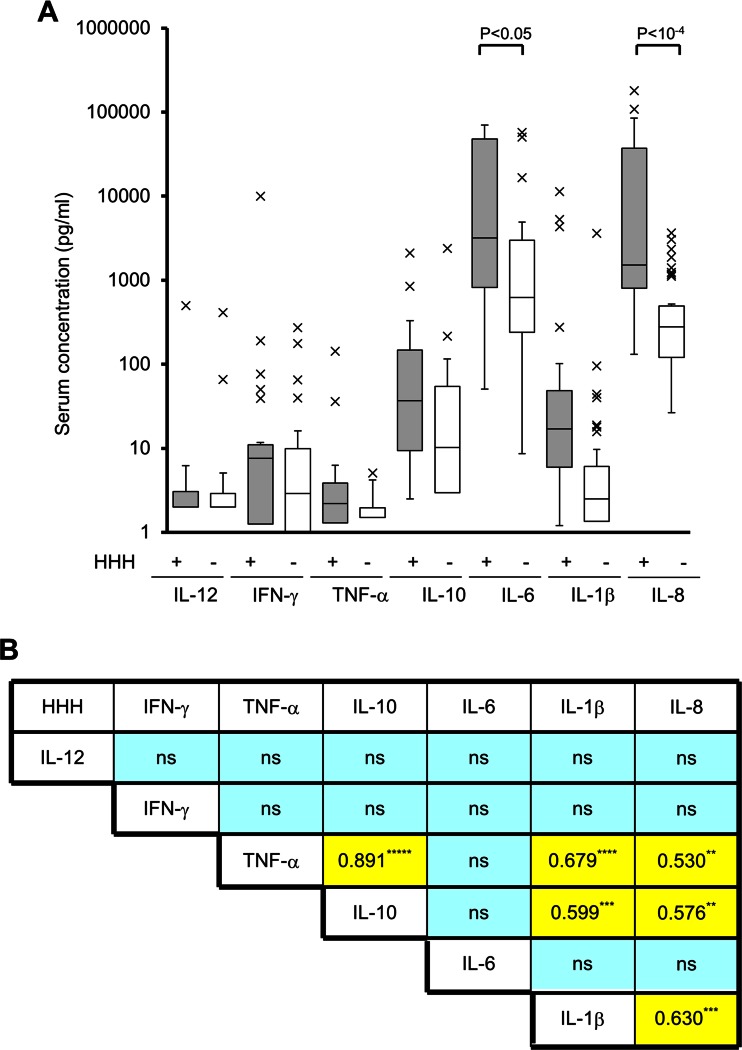
Serum cytokine analyses in patients with HHH. **a** Serum cytokine levels in patients with HHH. Serum was collected from the right atrium of each deceased patient and stored at −80 °C until analysis. The cytokine levels were measured by flow cytometry. *Grey bars* represent data derived from HHH patients, and *open bars* represent data from non-HHH patients. The data are shown by a *box and whisker plot. X*’s indicate statistical outliers. **b** Correlation coefficients among inflammatory cytokines and chemokines in HHH patients. Data represent the square of the correlation coefficient value. *, **, ***, ****, and ***** indicate statistically significant differences with *p* values <0.01, <10^−5^, <10^−6^, <10^−7^, and <10^−10^, respectively. *HHH*, histiocytic hyperplasia of hemophagocytosis; *ns*, not significant

HPS is induced by the simultaneous release of cytokines such as IFN-γ, TNF-α, IL-6, IL-1β, IL-10, IL-12, IL-8, and macrophage-colony stimulating factor [19]. In this study, IL-6 (20,662.2 ± 28,447.0 pg/ml vs. 4,262.5 ± 11,769.6 pg/ml, *p* < 0.001), IL-1β (749.8 ± 2,383.8 pg/ml vs. 95.5 ± 561.1 pg/ml, p < 0.05), and IL-8 (24,439.7 ± 42,733.5 pg/ml vs. 577.0 ± 816.1 pg/ml, *p* < 10^−6^) levels were significantly increased in HHH patients as compared with patients without HHH, while IL-12, IFN-γ, TNF-α, and IL-10 concentrations showed no statistically significant differences between the HHH and non-HHH groups (Fig. 3a, Table 3, and ESM Table 2).

**Table 3:** Clinical and physiological findings of patients with or without HHH

	HHH (*n* = 29)	No HHH (*n* = 41)	*P* ^a^ value
Organomegaly			
Hepatomegaly	16 (55.2 %)	15 (36.6 %)	
Splenomegaly	16 (55.2 %)	12 (29.3 %)	
Laboratory data			
WBC max (x10^3^/μl)	18.5 ± 13.6	17.4 ± 8.9	
WBC min (x10^3^/μl)	4.3 ± 7.4	5.7 ± 3.3	<0.05
Hb (g/dl)	7.2 ± 2.4	8.1 ± 2.5	
Plt (x10^4^/μl)	5.7 ± 10.8	11.6 ± 9.7	<0.05
AST (IU/l)	147 ± 170	766 ± 2,807	
Triglyceride (mg/dl)	239 ± 244	147 ± 89	
IL-12 (pg/ml)	19.0 ± 92.2	13.4 ± 64.4	
IFN-γ (pg/ml)	361.2 ± 1,854.2	17.3 ± 50.0	
TNF-α (pg/ml)	8.4 ± 26.7	1.4 ± 1.2	
IL-10 (pg/ml)	172.8 ± 407.8	78.9 ± 372.5	
IL-6 (pg/ml)	20,662.2 ± 28,447.0	4,262.5 ± 11,769.6	<0.05
IL-1β (pg/ml)	749.8 ± 2,383.8	95.5 ± 561.1	<0.05
IL-8 (pg/ml)	24,439.7 ± 42,733.5	577.0 ± 816.1	<10^−4^
Macrophages in BM cells (%)	25.2 ± 20.0	12.1 ± 4.0	<0.001

*HHH* histiocytic hyperplasia with hemophagocytosis, *WBC max* maximum number of WBC, *WBC min* minimum number of WBC

^a^Adjusted using the Holm method for multiple testing

Correlations among serum cytokine levels were also analyzed (Fig. 3b). In HHH patients, TNF-α was significantly correlated with IL-10 (*r* ^2^ = 0.819, *p* < 10^−10^), IL-1β (*r* ^2^ = 0.679, *p* < 10^−7^), and IL-8 (*r* ^2^ = 0.530, *p* < 10^−5^). Correlations were also detected between IL-10 and IL-1β (*r* ^2^ = 0.599, *p* < 10^−6^), IL-10 and IL-8 (*r* ^2^ = 0.576, *p* < 10^−5^), and IL-1β and IL-8 (*r* ^2^ = 0.630, *p* < 10^−6^).

### Multivariate risk factor analysis for HHH

To elicit the critical risk factors for HHH, logistic regression analysis was performed with variables that were significant in the univariate analysis. The presence of hematological diseases was associated with the development of HHH (odds ratio = 11.71; 95 % confidence interval (CI), 1.83–73.93, *p* < 0.01) (Table 4). Other factors associated with increased risk include 15 % or more of bone marrow macrophages (odds ratio = 9.42; 95 % CI, 2.14–41.58, *p* < 0.01), sepsis (odds ratio = 7.77; 95 % CI, 1.48–40.9, *p* < 0.05), and high serum IL-6 levels (odds ratio = 1.00; 95 % CI, 1.00–1.0001, *p* < 0.05).

**Table 4:** Multivariate risk analysis for HHH

	Odds ratio	95 % CI	*P* value
Hematological diseases	11.71	1.83–74.93	<0.01
Sepsis	7.77	1.48–40.9	<0.05
WBC (min)	1.12	0.94–1.33	0.20
IL-6	1.00	1.00–1.0001	<0.05
BM macrophages (≥15 % )	9.42	2.14–41.58	<0.01

*HHH* histiocytic hyperplasia with hemophagocytosis, *WBC (min)* minimum number of WBC, *BM* bone marrow, *CI* confidence interval

### Pathophysiological features of hypo-HHH

**Fig. 4:**
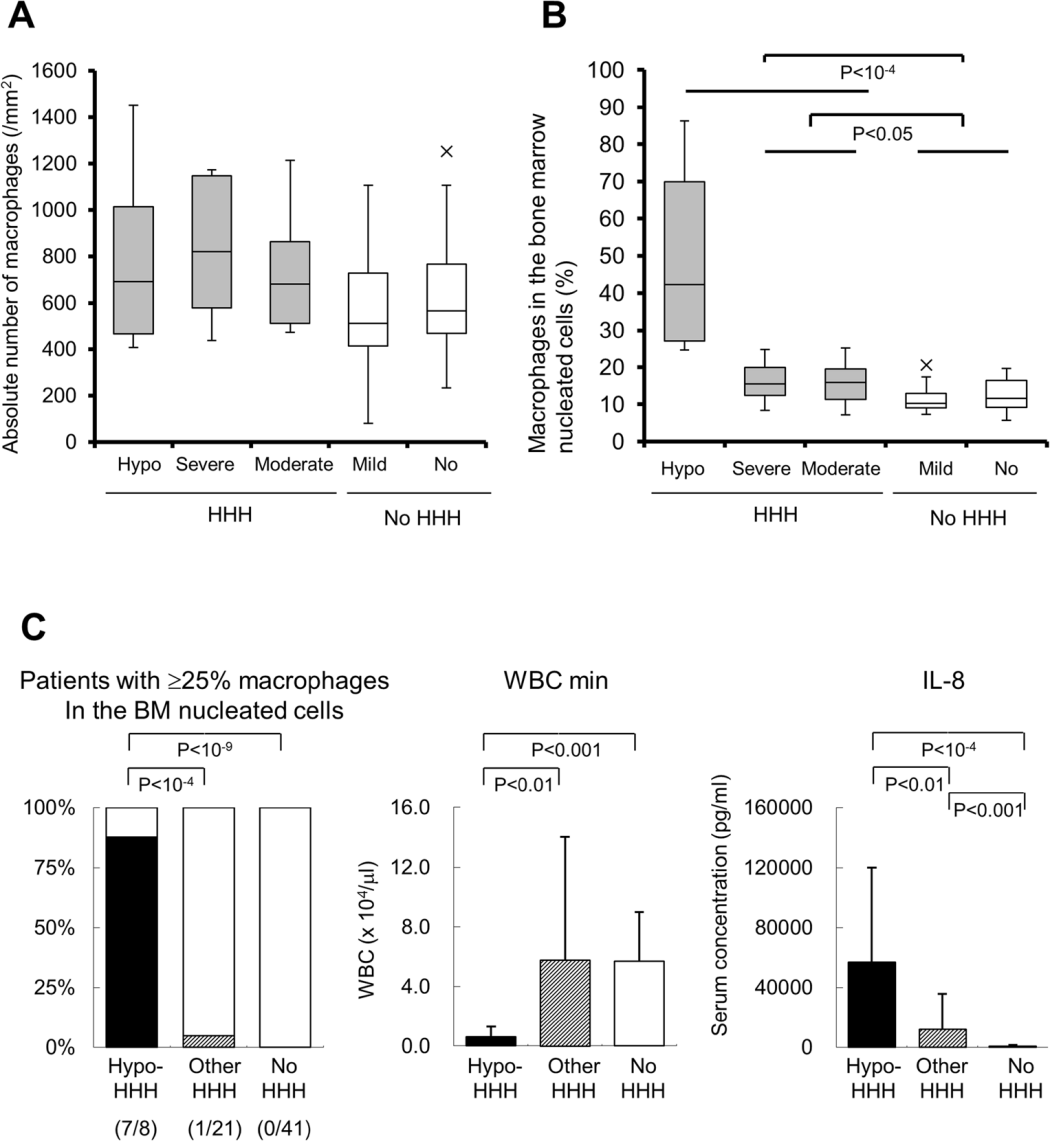
Features of hypo-HHH**. a** Absolute number of CD68-positive macrophages in the bone marrow in different types of HHH. **b** Percentage of macrophages in the bone marrow nucleated cells in each category. *Grey bars* represent data derived from HHH patients, and *open bars* represent data from non-HHH patients. The data are shown by a *box and whisker plot. X*’s indicate statistical outliers. **c** Characteristics of hypo-HHH in comparison with other-HHH and non-HHH patients. *Closed bars* represent data from hypo-HHH patients, *oblique bars* represent data from HHH patients, and *open bars* represent data from non-HHH patients. Data are presented as the mean ± standard deviation. *WBC min,* minimum number of white blood cells; *HHH*, histiocytic hyperplasia of hemophagocytosis; *hypo*-*HHH*, hypoplastic HHH

Most HPS patients have pancytopenia, which makes them susceptible to becoming immunocompromised. However, hypocellular bone marrow is rare in HPS patients, and there have been no previous reports of HHH with marrow hypoplasia. In the present study, we found eight HHH patients with both pancytopenia and hypocellular bone marrow. We therefore investigated the characteristics of patients with hypo-HHH (Table 5 and ESM Table 3). The absolute number of CD68-positive macrophages in the bone marrow was in the same range in each category (Fig. 4a). The percentage of macrophages in the bone marrow was significantly increased in hypo-HHH as compared with other types of HHH and non-HHH (Fig. 4b). Interestingly, the rate was similar between severe-HHH and moderate-HHH, and between mild-HHH and no-HHH. Thus, we compared the characteristics of hypo-HHH, other-HHH (including severe- and moderate-HHH), and no HHH with both mild-HHH and normal cohorts. As shown in Fig. 4c, seven of eight hypo-HHH patients had more than 25 % macrophages in the bone marrow. The hypo-HHH group had dramatically decreased minimum WBC counts (0.6 ± 0.7 × 10^3^/μl). Additionally, the IL-8 concentration was significantly increased in hypo-HHH patients as compared with other HHH patients (57,155.8 ± 62,993.6 pg/ml vs. 11,976.4 ± 24,010.6 pg/ml, *p* < 0.01, Fig. 4c). Six of the eight hypo-HHH patients received chemo-radiotherapies consisting of allogeneic peripheral blood stem cell transplantation (*n* = 2), chemotherapy (*n* = 3), and splenic irradiation (*n* = 1) (Table 6). The mean duration from the end of the final therapy to death was 79.8 ± 94.7 days (range, 20–270 days). Two of the eight hypo-HHH patients had not received myeloablative therapies, suggesting that the influence of chemo-radiotherapies was limited to the initiation of hypo-HHH.

**Table 5:** Features of patients with hypo-HHH or other HHH

	Hypo-HHH (*n* = 8)	Other HHH (*n* = 21)	*P* ^a^ value
Hematological diseases	7 (87.5 %)	7 (33.3 %)	
BM macrophages (≥25 %)	7 (87.5 %)	1 (4.8 %)	<0.001
WBC max (×10^3^/μl)	17.9 ± 16.6	18.7 ± 12.8	
WBC min (×10^3^/μl)	0.6 ± 0.7	5.8 ± 8.2	<0.05
Hb (g/dl)	6.1 ± 1.2	7.6 ± 2.6	
Plt (×10^4^/μl)	1.1 ± 1.1	7.5 ± 12.4	
Triglyceride (mg/dl)	408 ± 315	155 ± 150	
IL-12 (pg/ml)	1.8 ± 1.4	25.6 ± 108.3	
IFN-γ (pg/ml)	33.4 ± 65.8	486.0 ± 2,780.0	
TNF-α (pg/ml)	20.6 ± 3.8	49.5 ± 7.6	
IL-10 (pg/ml)	472.9 ± 708.8	58.5 ± 85.3	
IL-6 (pg/ml)	29,865.0 ± 33,572.3	17,156.4 ± 26,299.4	
IL-1β (pg/ml)	2,005.6 ± 4061.3	271.6 ± 1,145.6	
IL-8 (pg/ml)	57,155.8 ± 62,993.6	11,976.4 ± 24,010.6	<0.05

*HHH* histiocytic hyperplasia with hemophagocytosis, *WBC max* maximum number of WBC, *WBC min* minimum number of WBC

^a^Adjusted using the Holm method for multiple testing

**Table 6:** Days after the final chemo-radiotherapy of patients with hypo-HHH

No	Clinical diagnosis	Days after the final chemo-radiotherapy	Regimen
1	AML	51 days	CPA, TBI (Allo-PBSCT)
2	AML	23 days	CPA, TBI (Allo-PBSCT)
3	AML	55 days	Ara-C, MIT, VP-16
4	Malignant lymphoma	20 days	Ara-C, VP-16, DEX
5	Malignant lymphoma	60 days	L-ASP
6	Myelofibrosis	270 days	Splenic irradiation
7	Aplastic anemia	Not done	
8	Massive ascites	Not done	

*Allo-PBSCT* allogeneic peripheral blood stem cell transplantation, *AML* acute myelocytic leukemia, *Ara-C* cytarabine, *CPA* cyclophosphamide, *DEX* dexamethasone, *MIT* mitoxantrone hydrochloride, *L-ASP* l-asparaginase, *TBI* total body irradiation, *VP-16* etoposide

Regarding CD3 immunostaining and iron staining of bone marrow samples, the Fe staining detected hemophagocytic histiocytes. However, no significant association was obtained between the number of CD3-positive T lymphocytes and the severity of HHH or between the Fe-positive macrophage counts and the degree of HHH (ESM Figures 1 and 2).

## Discussion

HPS, which is characterized by the proliferation of activated macrophages with hemophagocytosis, is a rare syndrome [4, 6, 7, 20]. In contrast, HHH was detected in 41.4 % of this study cohort. Studies performed at two different university hospitals identified HHH in 35 of 107 deceased patients (32.7 %) [8] and 102 of 230 autopsies (44.3 %) [2]. In addition, the patients with mild HHH form the largest cohort in these previous studies. These data are consistent with our results, and together, they suggest that HHH is a common phenomenon in patients who die in major hospitals.

Two major categories of risk factors for HHH have been identified: (i) intrinsic factors, such as advanced malignancies, sepsis, and multi-organ failure; and (ii) extrinsic factors, such as blood transfusions and treatment intensity [2, 8, 21]. However, some controversial results have been obtained. In comparison with non-HHH patients, HHH patients had a significantly increased frequency of hematological diseases, hematological malignancies, and sepsis. Independent risk factors for HHH identified by logistic regression analysis were hematological diseases, ≥15 % bone marrow macrophages, sepsis, and high IL-6 levels. Hematological diseases were the most striking risk factor, and they have been implicated in severe immunodeficiency. Nevertheless, an underlying immunodeficient state at the onset of HHH and HPS seems underrepresented, unlike the documented infections for the most common setting of secondary HPS [22]. However, HHH developed preferentially in our hematological subjects with significantly decreased nadir WBC counts, particularly in hematological patients with hypo-HHH. Hematological patients with leukemia, malignant lymphoma, myelodysplastic syndrome, or aplastic anemia are potentially immunosuppressed, which would make them susceptible to severe infections such as pneumonia, febrile neutropenia, and bloodstream infections [15, 23]. Hematological disorder itself is not a condition of excessive inflammation; however, its associated risk of infections may lead to inflammation. The importance of severe infection for HHH may be validated by the other independent risk factors of sepsis and high IL-6 levels. In fact, sepsis is defined as the systemic response to infection and is involved in the systemic inflammatory response syndrome [11, 12]. IL-6 concentrations are proportional to the severity of infection [24]. The kinetics of inflammatory mediators after death remains unclear. Indeed, few studies have provided sequential analyses of inflammatory cytokine levels after death. As for the IL-6 and IL-1β concentrations, the mean levels tend to gradually increase at 24 h after death in both septic patients and normal cohorts, but several cases have shown decreased levels after death [25, 26]. Because our autopsies were performed within 24 h of death (3.0 ± 2.6 h; range, 1–15.5 h), postmortem modifications are assumed to be slight. The results of previous studies together with the present results suggest that underlying diseases characterized by excessive inflammatory conditions are the most striking initiators of HHH.

The median time from onset to diagnosis of hemophagocytic lymphohistiocytosis is 3.5 months [19], and the activated inflammatory cytokines often persist for 100 days [5]. In addition, the impairment of the Th1 and Th2 balance promotes secretion of Th1 cytokines such as IFN-γ, IL-4, IL-12, and IL-18, followed by the elevation of pro-inflammatory TNF-α, IL-1β, and IL-6 cytokines, IL-8 chemokine, and anti-inflammatory IL-10 cytokine [5, 10, 19]. However, the profiles of inflammatory cytokines have not been previously analyzed in HHH patients. We found that HHH patients had significantly higher levels of IL-1β, IL-6, and IL-8 as compared with patients without HHH. Several mediators including TNF-α, IL-1β, IL-8, and IL-10 showed strong positive correlations, indicating that the secreted cytokines were closely associated. The released cytokines were similar to the cytokine profiles described for septic shock, severe febrile neutropenia, and in vitro sepsis model experiments that used peripheral blood monocytes [27–29]. Moreover, significant correlations are noted among inflammatory cytokines in severe inflammatory diseases [28, 29]. These findings suggest that a series of inflammatory cytokine releases was a feature of HHH as well as other severe inflammatory diseases and that monocytes and macrophages were responsible for the production of these cytokines. On the other hand, the levels of IFN-γ did not show a statistically significant difference between the HHH and non-HHH patients. The levels of IFN-γ decrease rapidly with improvements in the HPS syndrome [5, 19], suggesting that the IFN-γ concentration had already attenuated at the time of death.

HHH patients have been reported to have normocellular bone marrow, but we identified eight HHH patients with hypocellular bone marrow. Hypo-HHH was characterized by an increased rate of migrated macrophages in the bone marrow, severe leukocytopenia, and high serum IL-8 levels; however, there was no increase in the absolute number of macrophages, indicating that the functional activation of macrophages resulted in the engulfment of bone marrow hematopoietic cells. High IL-8 concentrations may be associated with the seriousness of HHH. Indeed, the chemokine concentration was significantly increased according to the severity of HHH. Even in the patients with mild HHH, the IL-8 concentration was greater than that in the normal cohort (714.2 ± 649.0 pg/ml vs. 505.8 ± 893.7 pg/ml, *p* < 0.05), although their macrophage counts were in the same range. Conversely, the minimum WBC count of mild HHH was significantly lower than that of non-HHH (4.2 ± 2.9 × 10^3^/μl vs. 6.5 ± 3.2 × 10^3^/μl, *p* < 0.05). IL-8 is a CXC chemokine produced by monocytes and macrophages that attracts neutrophils to inflamed sites for phagocytosis and killing of pathogens [27, 30]. The IL-8 concentrations are inversely correlated with leukocytopenia, and the peak value is seen during the neutropenic phase of hematopoietic stem cell transplantation [31]. These findings suggest that the high IL-8 level in hypo-HHH is the consequence of severe leukocytopenia due to excessive activation of bone marrow macrophages.

Strauss et al. described HPS as the most severe variant of HHH [8]. However, none of the HHH patients in their study or in our study fulfills the diagnostic criteria for HPS. Indeed, HPS and HHH have hypercytokinemia in common, but HPS is a syndrome with defined clinical and laboratory features, one of which is the occurrence of increased histiocytes with hemophagocytosis. By contrast to HPS, the development of HHH is most often a multifactorial process rather than one related to a single underlying condition [2]. Therefore, HHH should be considered as a different disease entity from HPS. Additionally, we found a new feature of HHH in that the absolute number of BM histiocytes was in a similar range in the presence or absence of HHH, indicating that the essential feature of HHH is the augmentation of hemophagocytic histiocytes rather than histiocytic hyperplasia. Hence, we propose a tentative definition of this disorder as “polyhemophagocytosis (PHP)” to avoid confusion with HPS (Table 7) [2, 8].

**Table 7:** A tentative definition of “polyhemphagocytosis (PHP)”

Prerequisite condition	Inai	Strauss	Suster
≥1–3 hemophagocytic cells/HPF in BM, lymphocytes, or spleen	Yes	Yes	Yes
Promising situations			
Underlying diseases			
Sepsis	Yes	Yes	Yes
≥ 15 % BM macrophages	Yes	NE	NE
Recent transfusion	NE	Yes	Yes
Hematological malignancies and aplastic anemia	Yes	NE	NE
Cause of death			
ARDS/sepsis/septic shock	Yes	Yes	NE
Exclusion			
Definitive HPS/HLH	Yes	Yes	Yes

*ARDS* acute respiratory distress syndrome, *BM* bone marrow, *HLH* hemophagocytic lymphohistiocytosis, *HPF* high-power field, *HPS* hemophagocytic syndrome, *NE* not evaluated

In conclusion, we found that conditions of excessive inflammation, such as hematological disorders and sepsis, appear to be the strongest initiators of HHH development. In addition, the development of HHH was also associated with increased IL-6 and IL-8 concentrations. Although further investigations of HHH are needed, insights into HHH can be gained from the analysis of data from the present study.

## Electronic supplementary material

Below is the link to the electronic supplementary material.

Supplemental Table 1 (PDF 72 kb)

Supplemental Table 2 (DOC 47 kb)

Supplemental Table 3 (DOC 37 kb)

Supplemental Figure 1 (GIF 40 kb)

High Resolution Image (TIFF 219 kb)

Supplemental Figure 2 (GIF 144 kb)

High Resolution Image (TIFF 1473 kb)
