# Supplementary material for: Risk factor analysis for bone marrow histiocytic hyperplasia with hemophagocytosis: an autopsy study
Source: Virchows Arch. 2014 May 23;465(1):109–18. doi: 10.1007/s00428-014-1592-8 (PMC4077255; doi:10.1007/s00428-014-1592-8)
Supplement: Supplementary file 1 — (PDF 72 kb) [file 428_2014_1592_MOESM1_ESM.pdf]

Supplemental Table 1. Detailed information of the underlying conditions in each patient

No	Age	Sex	HHH	Bone marrow cellularity	Malignancy	Clinical diagnosis	Major metastatic sites	Major complication	Sepsis	DIC	Shock
1	53	F	Hypo-	Hypocellular	+	AML		sepsis (Fusarium, CMV, bacteria)	+	+	-
2	56	M	Hypo-	Hypocellular	+	AML		sepsis (fungus, CMV, bacteria)	+	+	-
3	66	M	Hypo-	Hypocellular	+	AML		lobal pneumonia	-	-	-
4	66	F	Hypo-	Hypocellular	+	Malignant lymphoma		sepsis, brain abscess (bacterial)	+	+	+
5	54	M	Hypo-	Hypocellular	+	Malignant lymphoma	brain, liver, spleen, kidney	aspiration pneumonia	-	+	-
6	78	M	Hypo-	Hypocellular	+	Myelofibrosis		pneumonia	-	+	-
7	59	M	Hypo-	Hypocellular	-	Aplastic anemia		TRALI	-	+	+
8	53	M	Hypo-	Hypocellular	-	Massive ascites		enterocolitis, pneumonia, sepsis	+	+	-
9	75	M	Severe	Hypercellular	+	AML	liver, spleen, adrenal gland, lymph nodes	pneumonia (Aspergillosis), sepsis	+	+	-
10	79	F	Severe	Hypercellular	+	AML		infectious endocarditis, sepsis	+	-	+
11	60	F	Severe	Normocellular	+	Malignant lymphoma		sepsis (CMV)	+	+	+
12	71	M	Severe	Hypercellular	+	Malignant lymphoma	bone marrow, liver, spleen, pancreas		-	+	-
13	76	M	Severe	Hypercellular	+	MDS	spleen	pulmonary abscess, prostatitis	-	+	-
14	65	F	Severe	Normocellular	-	Microscopic polyangiitis		sepsis (fungus)	+	+	+
15	55	F	Severe	Normocellular	+	Ovarian cancer	ovary, bladder, peritoneum		-	-	-
16	64	F	Severe	Hypercellular	+	External genital cancer	skin, peritoneum, lymph nodes	pneumonia	-	+	-
17	53	M	Severe	Hypercellular	+	Rectal cancer	brain, lung, liver, kidney, adrenal gland, skin, lymph nodes		-	-	-
18	59	M	Severe	Hypercellular	+	Bile duct cancer	lung, pleura, liver, pancreas, peritoneum, bone marrow, lymph nodes		-	-	-
19	65	F	Severe	Slightly hypercellular	-	Acute subdural hemorrhage		pneumonia	-	+	-
20	53	M	Moderate	Hypercellular	+	AML	liver, spleen, kidney, testis	sepsis, tumor lysis syndrome	+	+	+
21	70	F	Moderate	Hypercellular	+	AML	liver, spleen, lymph nodes	lobal pneumonia	-	+	-
22	68	F	Moderate	Slightly hypocellular	+	Gallbladder cancer	brain, lung, liver, adrenal gland, lymph nodes	sepsis	+	+	-
23	91	M	Moderate	Normocellular	-	Hemorrhagic gastric ulcer		sepsis	+	-	+
24	74	M	Moderate	Normocellular	-	Interstitial pneumonia		interstitial pneumonia	-	-	-
25	70	M	Moderate	Hypercellular	-	Liver cirrhosis			-	-	+
26	69	F	Moderate	Normocellular	+	Colon cancer	lung, diaphragma, liver, peritoneum, rectum	pyelonephritis, pneumonia, sepsis	+	-	-
27	59	M	Moderate	Normocellular	+	Colon cancer	liver, peritoneum	pan-pertnitis, sepsis	+	-	+
28	54	F	Moderate	Hypercellular	+	Breast cancer	brain, lung, heart, liver, spleen, bone marrow, lymph nodes	aspiration pneumonia	-	+	-
29	83	F	Moderate	Normocellular	+	Gastric cancer	lung, pancreas, colon, peritoneum, lymph nodes		-	-	-
30	73	M	Mild	Hypercellular	+	AML			-	-	+
31	68	F	Mild	Hypocellular	+	Malignant lymphoma	skin, adrenal gland, lymph nodes	pneumonia, acute tubular necrosis	-	-	-
32	70	F	Mild	Hypercellular	+	Malignant lymphoma	bone marrow, lung, liver, spleen, adrenal gland, kidney, uterus		-	+	-
33	74	F	Mild	Hypercellular	+	Malignant lymphoma	brain, bone marrow, heart, lung, liver, spleen, kidney, bladder		-	+	-
34	79	F	Mild	Hypercellular	+	MDS	heart, lung, esophagus, liver, spleen, kidney, lymph nodes		-	+	-
35	56	M	Mild	Hypercellular	+	Gall bladder cancer	lung, liver, spleen, peritoneum, adrenal gland, lymph nodes	pneumonia (fungus)	-	+	-
36	79	M	Mild	Hypercellular	+	Lung cancer	bone marrow, pleura, kidney, adrenal gland, skin, lymph nodes	pneumonia	-	-	-
37	53	M	Mild	Normocellular	-	Hypertrophic cardiomyopathy		prostatitis	-	-	-
38	79	M	Mild	Normocellular	-	Pneumonia		pneumonia	-	-	-

39	63	M	Mild	Normocellular	+	Hepatoma	lung, adrenal gland		-	-	+
40	74	F	Mild	Normocellular	+	Ovarian cancer	lymph nodes	pneumonia	-	-	-
41	52	M	Mild	Normocellular	+	Gingival cancer	lung, lymph nodes	pulmonary abscess	-	-	-
42	71	M	Mild	Normocellular	+	Glioblastoma		pneumonia	-	-	-
43	85	F	Mild	Hypercellular	+	Cervical cancer	bladder		-	-	-
44	73	M	-	Normocellular	+	Lung cancer	lung	sepsis (MRSA)	+	+	+
45	71	M	-	Hypercellular	+	Lung cancer	lung, liver, bone marrow, adrenal gland, kidney, lymph nodes	acute tubular necrosis	-	+	-
46	90	F	-	Hypercellular	+	Colon cancer	lymph nodes	pneumonia, pyelonephritis	-	-	-
47	87	F	-	Normocellular	+	Pancreas cancer	bile duct, duodenum		-	+	-
48	50	M	-	Hypocellular	+	Hepatoma			-	-	+
49	81	M	-	Hypocellular	+	Hepatoma			-	-	+
50	0	M	-	Normocellular	-	Infectious enteritis		sepsis (Acinetobacter)	+	-	+
51	76	F	-	Normocellular	-	Diabetes mellitus		tuberculosis, colitis	-	+	-
52	84	M	-	Normocellular	-	Viral pneumonia		pneumonia (CMV)	-	+	-
53	42	F	-	Hypercellular	-	MELAS		pneumonia	-	+	-
54	52	F	-	Normocellular	-	MELAS			-	+	-
55	51	M	-	Normocellular	-	Alcoholic liver cirrhosis			-	+	+
56	68	M	-	Hypocellular	-	Diabetes mellitus			-	-	-
57	94	F	-	Normocellular	-	Pneumonia			-	-	-
58	68	M	-	Normocellular	-	Diffuse panbronchiolitis			-	-	-
59	42	M	-	Normocellular	-	Osteochondrodysplasia			-	-	-
60	65	F	-	Normocellular	+	Pharyngeal cancer		sepsis	+	-	-
61	67	M	-	Slightly hypercellular	+	Hypopharyngeal cancer	lung, liver, lymph nodes	sepsis	+	+	+
62	74	F	-	Slightly hypercellular	+	Renal cancer	brain, bone marrow, liver, spleen, kidney, adrenal gland, lymph nodes	pneumonia	-	-	-
63	87	F	-	Normocellular	+	Cervical cancer	en bloc of pelvic organs	pneumonia, pyelonephiritis	-	-	-
64	81	M	-	Hypercellular	+	Lung cancer	bone marrow, lung, liver, lymph nodes	aspiration pneumonia	-	-	-
65	52	F	-	Hypercellular	+	Colon cancer	bone marrow, lung, liver, adrenal gland, kidney, lymph nodes	pan-peritnitis	-	-	-
66	78	M	-	Hypercellular	+	Gastric cancer	peritoneum		-	-	-
67	64	M	-	Hypercellular	+	Gastric cancer	bone marrow, lung, liver, duodenum, lymph nodes		-	-	-
68	74	F	-	Normocellular	+	Ovarian cancer	peritoneum		-	-	-
69	35	M	-	Hypercellular	-	Infectious endocarditis		sepsis	+	+	-
70	73	F	-	Normocellular	-	Subarachnoid hemorrhage		pneumonia	-	-	-

Abbreviations; AML; acute myelocytic leukemia, CMV; cytomegalovirus, MDS; myelodysplastic syndrome, MELAS; myopathy, encephalopathy, lactic acidosis and stroke-like episode, MRSA; methicillin-resistant staphylococcus aureus, TRALI; transfusion-related acute lung injury
