# Supplementary material for: Risk factor analysis for bone marrow histiocytic hyperplasia with hemophagocytosis: an autopsy study
Source: Virchows Arch. 2014 May 23;465(1):109–18. doi: 10.1007/s00428-014-1592-8 (PMC4077255; doi:10.1007/s00428-014-1592-8)
Supplement: Supplementary file 2 — (DOC 47 kb) [file 428_2014_1592_MOESM2_ESM.doc]

**Supplemental Table 2. P values before and after the adjustment by the Holm method for multiple testing in patients with HHH**

| **Univariate analysis** | ***P*** | ***P**** |
| --- | --- | --- |
| IL-8 | 0.00000099 | 0.00002772** |
| Increase of BM macrophages | 0.000016 | 0.000432** |
| IL-1β | 0.00044 | 0.01144** |
| ARDS/sepsis/septic shock (cause of death) | 0.00046 | 0.0115** |
| Plt | 0.00062 | 0.01488** |
| IL-6 | 0.000679 | 0.015617** |
| Hematological diseases | 0.00083 | 0.01826** |
| WBC min | 0.0012 | 0.0252** |
| Sepsis (underlying condition) | 0.00209 | 0.0418** |
| Hematological malignancy | 0.00209 | 0.0418** |
| TNF-α | 0.00317 | 0.05706 |
| IL-10 | 0.00508 | 0.08636 |
| DIC | 0.0209 | 0.3344 |
| Infection | 0.0227 | 0.3405 |
| Splenomegaly | 0.0293 | 0.4102 |
| Solid malignancy | 0.0604 | 0.7852 |
| Hb | 0.1 | 1.2 |
| Hepatomegaly | 0.123 | 1.353 |
| IFN-γ | 0.25 | 2.5 |
| Shock | 0.268 | 2.412 |
| Age | 0.302 | 2.416 |
| Triglyceride | 0.36 | 2.52 |
| Malignancy (all cases) | 0.368 | 2.208 |
| Pneumonia | 0.537 | 2.685 |
| Sex | 0.939 | 3.756 |
| IL-12 | 0.94 | 2.82 |
| AST | 0.96 | 1.92 |
| WBC max | 0.96 | 1.92 |

ARDS, acute respiratory distress syndrome; BM, bone marrow; DIC, disseminated intravascular coagulation; HHH, histiocytic hyperplasia with hemophagocytosis; WBC max; maximum number of white blood cells; WBC min, minimum number of white blood cells; *Adjusted by using the Holm method for multiple testing, ** Finally, decided as statistically significant.
