# Supplementary material for: Risk factor analysis for bone marrow histiocytic hyperplasia with hemophagocytosis: an autopsy study
Source: Virchows Arch. 2014 May 23;465(1):109–18. doi: 10.1007/s00428-014-1592-8 (PMC4077255; doi:10.1007/s00428-014-1592-8)
Supplement: Supplementary file 3 — (DOC 37 kb) [file 428_2014_1592_MOESM3_ESM.doc]

**Supplemental Table 3. P values before and after the adjustment by the Holm method for multiple testing in cases with hypo-HHH**

| **Univariate analysis** | ***P*** | ***P**** |
| --- | --- | --- |
| BM macrophages (≥25% ) | 0.00000837 | 0.00011718** |
| WBC min | 0.00179 | 0.02327** |
| IL-8 | 0.00399 | 0.04788** |
| Triglyceride | 0.00506 | 0.05566 |
| IL-10 | 0.00629 | 0.0629 |
| Hematological diseases | 0.00908 | 0.08172 |
| Plt | 0.0672 | 0.5376 |
| IL-6 | 0.0749 | 0.5243 |
| TNF-α | 0.113 | 0.678 |
| IL-1β | 0.118 | 0.59 |
| Hb | 0.2457 | 0.9828 |
| WBC max | 0.661 | 1.983 |
| IFN-γ | 0.826 | 1.652 |
| IL-12 | 0.922 | 0.922 |

BM, bone marrow; HHH, histiocytic hyperplasia with hemophagocytosis; WBC max, maximum number of white blood cells; WBC min, minimum number of white blood cells; *Adjusted by using the Holm method for multiple testing. ** Finally, decided as statistically significant.
