# Supplementary figures and images for: Risk factor analysis for bone marrow histiocytic hyperplasia with hemophagocytosis: an autopsy study
Source: Virchows Arch. 2014 May 23;465(1):109–18. doi: 10.1007/s00428-014-1592-8 (PMC4077255; doi:10.1007/s00428-014-1592-8)

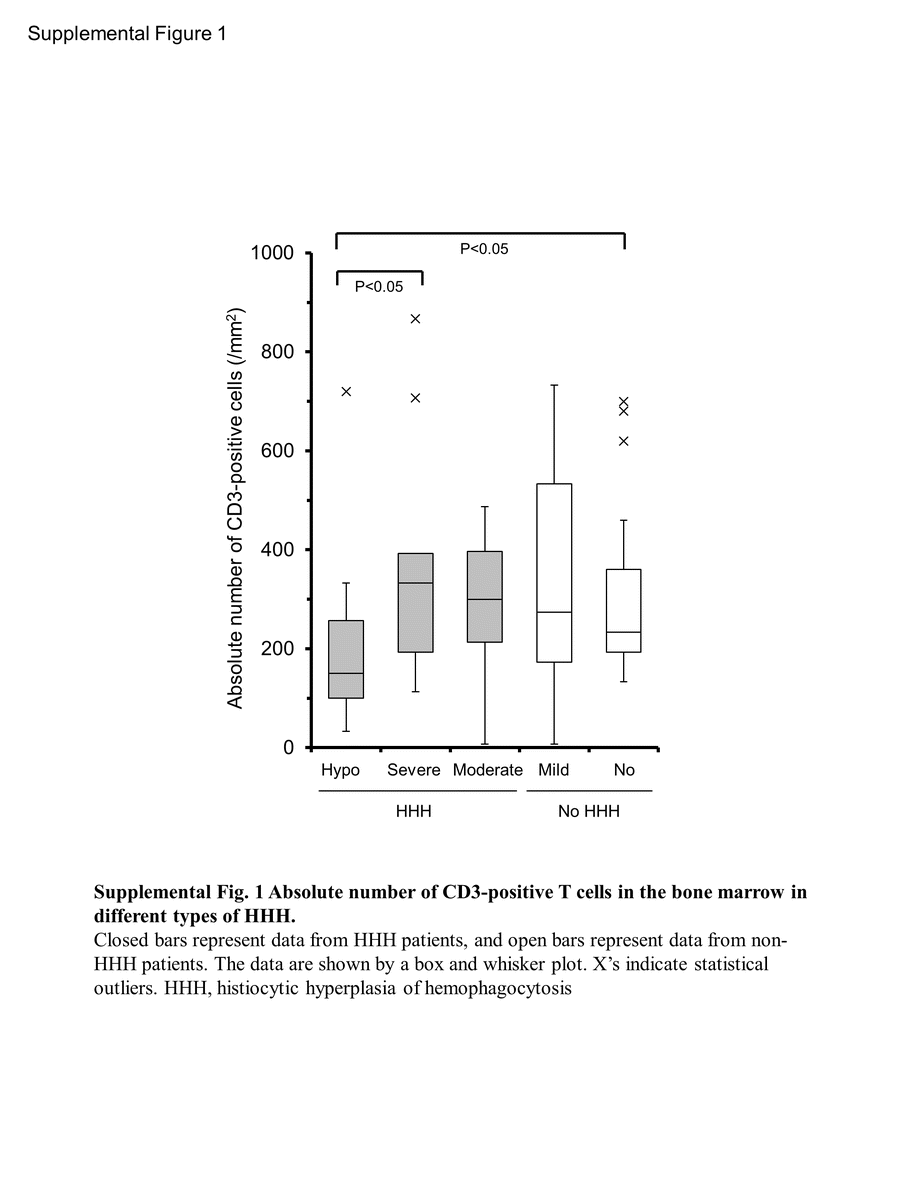

Supplement: Supplementary file 4 — (GIF 40 kb) [file 428_2014_1592_Fig5_ESM.gif]

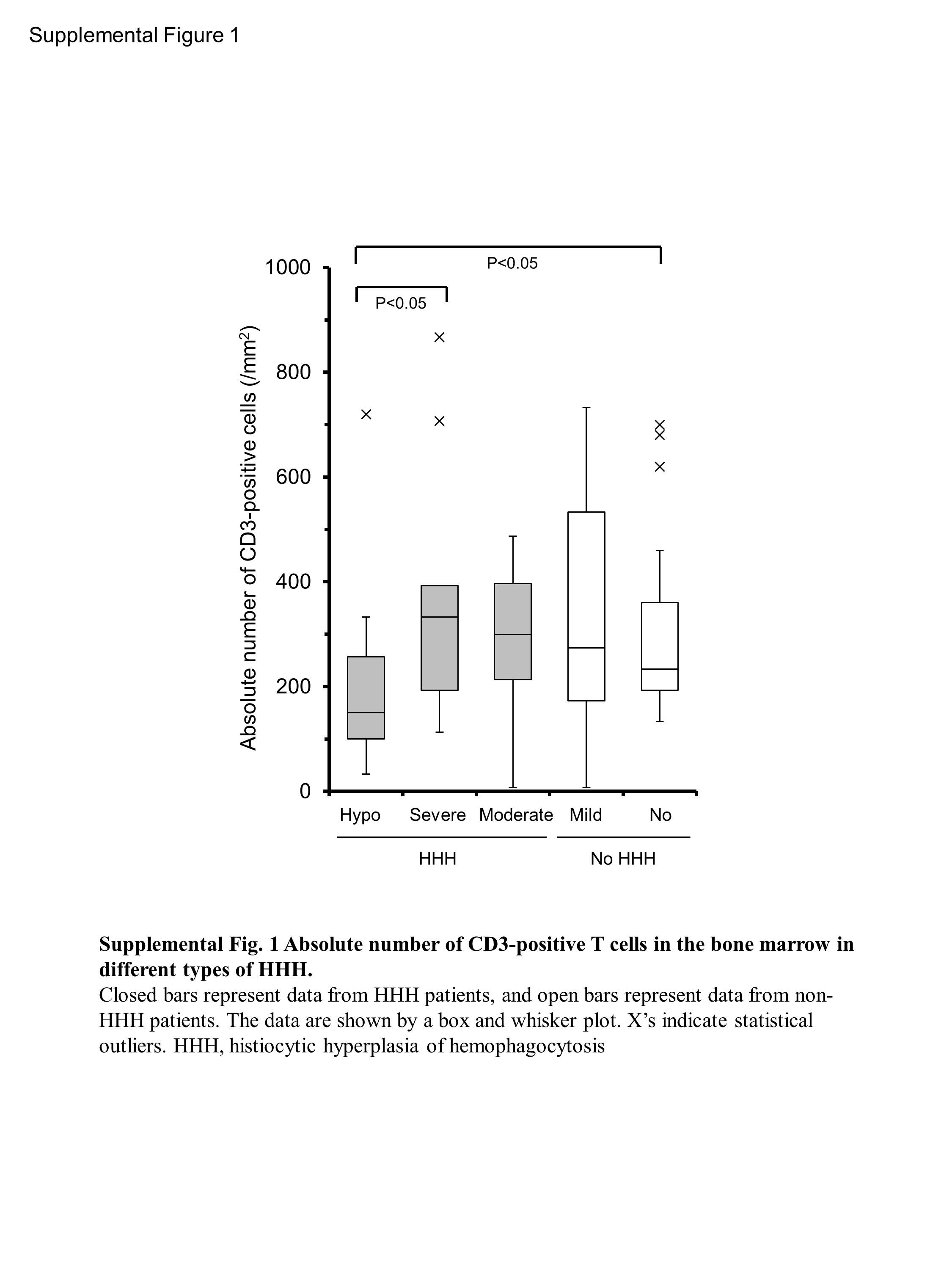

Supplement: Supplementary file 5 — High Resolution Image (TIFF 219 kb) [file 428_2014_1592_MOESM4_ESM.tif]

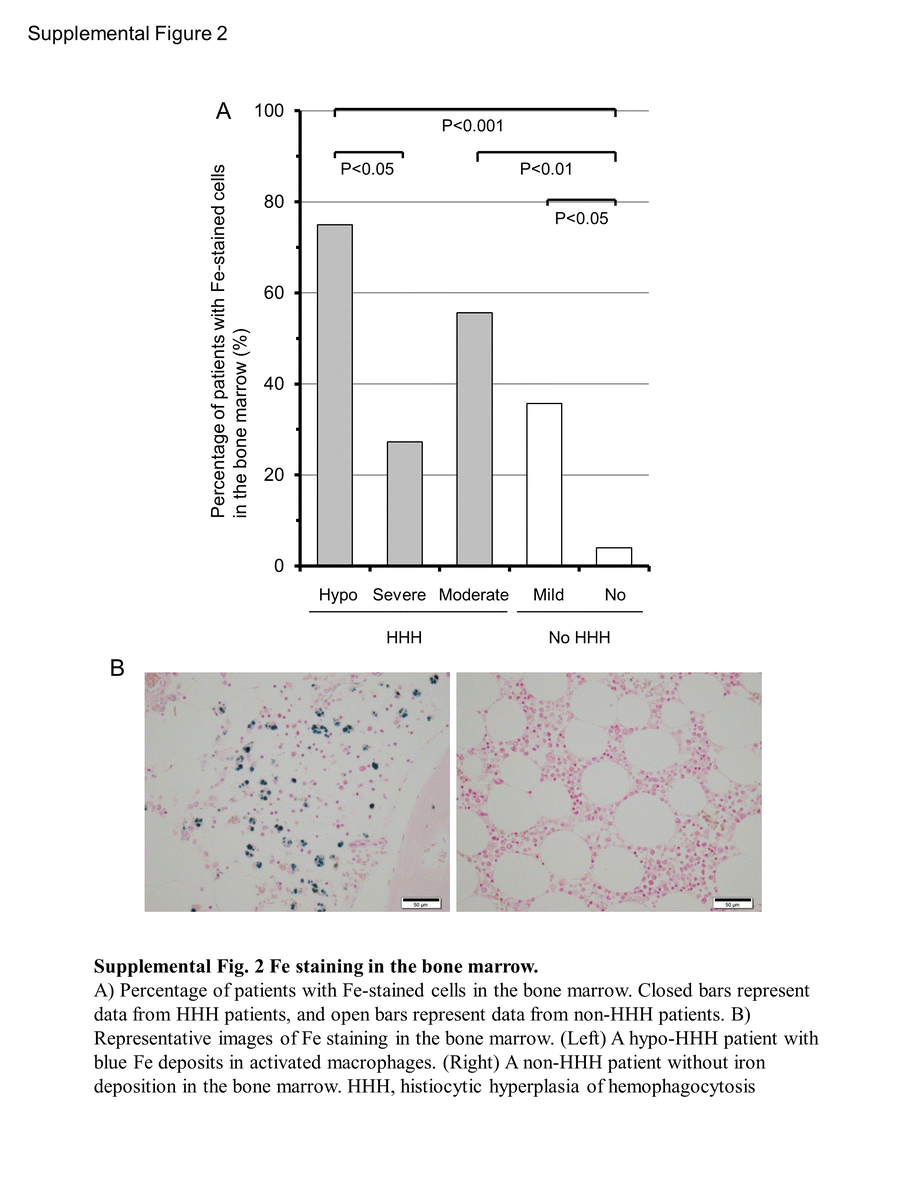

Supplement: Supplementary file 6 — (GIF 144 kb) [file 428_2014_1592_Fig6_ESM.gif]

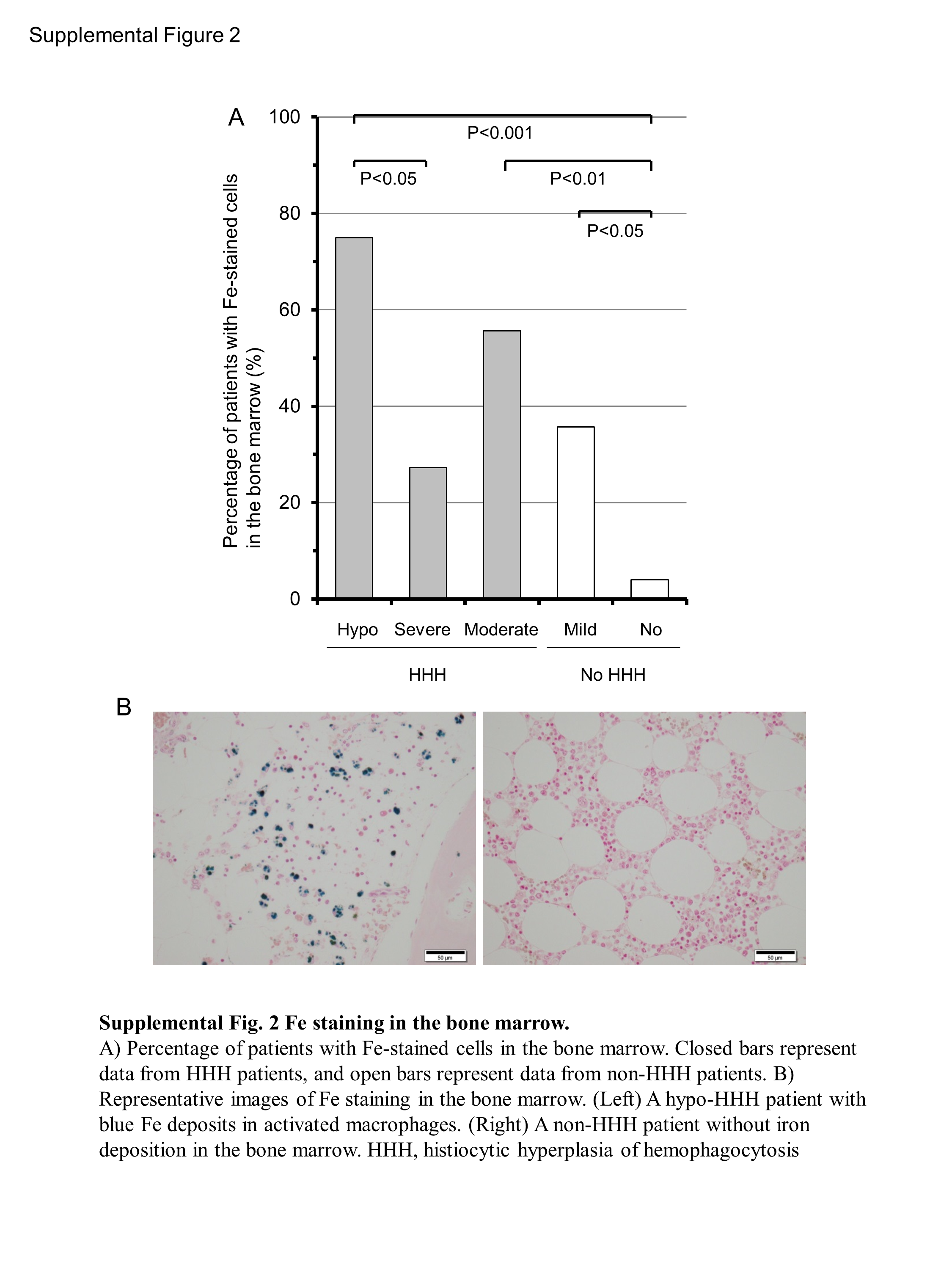

Supplement: Supplementary file 7 — High Resolution Image (TIFF 1473 kb) [file 428_2014_1592_MOESM5_ESM.tif]
